# Characterizing nrDNA ITS1, 5.8S and ITS2 secondary structures and their phylogenetic utility in the legume tribe Hedysareae with special reference to *Hedysarum*

**DOI:** 10.1371/journal.pone.0283847

**Published:** 2023-04-12

**Authors:** Haniyeh Nafisi, Akram Kaveh, Shahrokh Kazempour-Osaloo

**Affiliations:** Faculty of Biological Sciences, Department of Plant Biology, Tarbiat Modares University, Tehran, Iran; Chinese Academy of Medical Sciences and Peking Union Medical College, CHINA

## Abstract

This is the first study to systematically evaluate rRNA secondary structures of Hedysareae with an emphasis on *Hedysarum*. ITS2 and 5.8S regions of the genus shared a common secondary structure with a four-fingered central loop, whereas ITS1 possessed five distinct structures. The secondary structural features of the two regions provided advantageous data for clades, species groups, and closely related species. Hemi-CBCs were mostly observed in the reconstruction of species groups, and Nsts, mostly between closely related species. The investigations showed that ITS1 varied more than ITS2 in length, GC content, and most of the diversity indices within the tribe. Maximum likelihood analyses of the synchronized sequence-structure tree of ITS1 were performed. The accuracy and phylogenetic signals of ITS1 were higher than ITS2. The similar GC content, and no CBC, in both spacers, fortified the close relationship of CEGO and *H*. sections *Stracheya* and *Hedysarum* clades in the synchronized sequence-structure tree topology of ITS1. In both regions, no inter-generic CBCs were detected inside the CEGO clade and the inter-sectional level of *Hedysarum*. But, in the ITS2 region, a CBC was detected between *H*. section *Multicaulia*, and *Taverniera* versus *H*. sections *Hedysarum*, and *Stracheya*. The lowest inter-sectional genetic distance and structural features were found between *H*. sect. *Hedysarum* and *H*. sect. *Stracheya* clades in the ITS2 region.

## 1 Introduction

*Hedysarum* L., the largest genus of the tribe Hedysareae (Fabaceae), is distributed in temperate Eurasia, NW Africa, W Canada to W & Central U.S.A [1–3]. The non-monophyletic identity of *Hedysarum* in the nuclear tree topology, in contrast to plastid topology, has been confirmed in previous molecular phylogenetic studies [1,4,5]. Liu et al. [6], run the chloroplast capture hypothesis via introgression as the best explanation for the incongruence. Phylogenetic analyses of nrDNA ITS based on Nafisi et al., [5] retrieved *Hedysarum* with three well-supported clades redefined as three sections of *Hedysarum*, *Stracheya*, and *Multicaulia*. *Hedysarum*. sect. *Hedysarum* (clade H) formed a clade relationship with *Ebenus* and *Taverniera*. Subsequently, the CEGO clade (*Greuteria*, *Eversmannia*, *Corethrodendron*, and *Onobrychis*; Liu et al., [6]) diverged. At the tip, *H*. sect. *Stracheya* (clade S) and *H*. sect. *Multicaulia* are located in a hardly supported clade. *Hedysarum* sect. *Multicaulia* in turn fell into two well-supported clades. The small lineage corresponds to the *H*. sect. *Multicaulia* subsect. *Multicaulia* (clade M), and the well-rich lineage of southwest Asia species defined as *H*. sect. *Multicaulia* subsect. *Crinifera* (clade C) [5]. In all directed phylogenetic studies, the closest genus to *Hedysarum* remained provisional, and the relationship of six recently defined species groups in the *H*. sect. *Multicaulia* subsect. *Crinifera* [7] remained unresolved in both plastid and nuclear tree topologies. Moreover, we need a cutting-edge tool to handle taxonomic studies in delineating newly reported species.

The internal transcribed spacer (ITS) region of ribosomal RNA is the most commonly sequenced locus used in plant phylogenetic investigations from the tribal to the species level and shows high levels of interspecific divergence as a barcode [8].

The Internal Transcribed Spacer 2 (ITS2) is a highly divergent and fast-evolving part of the eukaryotic nuclear-encoded rRNA operon located between the 5.8S and 28S rRNA genes, frequently used as a mini barcode for medicinal plant species [9–11]. Despite the high mutation rate, ITS2 forms a highly conserved secondary structure to catalyze the maturation of the ribosomal RNA [12–17]. The structure of 4-fingered central loop, UGGU motif near the apex of Helix III (longest Helix), and U-U mismatch in Helix II are considered the characteristics of a conserved ITS2 secondary structure [17]. It keeps excellence in the improvement of the accuracy and robustness of phylogenetic tree reconstruction by the inclusion of secondary structure [18–28] and by comparing the structures [11,29–31].

In addition, the presence of any compensatory base change (CBC) in the conserved regions of helices 2 and 3 of ITS2 revealed a correlation with incompatibility/ ability to sexually cross [32,33]. Detections about the effectiveness of ITS2 in distinguishing cryptic/pseudo-cryptic species via CBCs [34–36], resulting in its frequent use to resolve lower taxonomic affinities of copious primitive eukaryotes, such as diatoms and alveolates [2,37–43], fungi [7,44,45], algae [34,46–48] some cases of terrestrial plants [22,49–51] and zoophytes [52–54]. Furthermore, following Torres-Suárez [55], utilizing hemi-CBCs (hCBCs) to assess relationships at the population and species levels and Karpenko et al., [51], using nonstructural substitutions (Nsts) and hCBCs at the species level, Ozgişi [27] applied both for species delimitation.

The Internal Transcribed Spacer 1 (ITS1) is the noncoding region between 18S rRNA and 5.8S genes. It has been used as a universal fungal barcode for quick general analyses of diversity [56]. Typically, it is more variable than ITS2, and its secondary structure indicates no definite core pattern like that of ITS2 [7,57–60]. In some cases, conservation at the structural level despite dramatic nucleotide sequence variation is observed, involving the processing events during the maturation of rRNAs [4,12,13,61–63]. Whereas in some cases were more variable in ITS2 and revealed as a more suitable marker for operational taxonomic richness [59,64], Nilsson et al., [59] and Monard et al., [58] observed a correlation between the variability of ITS1 vs. ITS2, suggesting that the two regions do not evolve separately and established as complementary. Meanwhile, Mello et al., [65] and Blaalid [66] introduced both markers as suitable DNA metabarcoding.

This is the first study to systematically investigate the potential use of ITS1, 5.8S, and ITS2 consensus secondary structure prediction toward species identification of *Hedysarum* and affinities. The CBC species concept has not yet been tested for members of the tribe Hedyasreae. Since the morphological variations cause mistakes when identifying the species, the aim herein was to test if the CBCs, hCBCs, and Nsts, are useful for distinguishing tribe members. Furthermore, to test if a sequence-structure approach for both datasets enhances resolution or provide additional insights into Hedysareae phylogeny when compared to a sequence-only approach.

## 2 Materials and methods

### 2.1 Datasets

Internal transcribed spacer sequences of *Hedysarum* species characterized by multi-loci phylogeny available from GenBank were retrieved. Furthermore, 18 taxa were newly sequenced. The accessions with the same sequence and without complete length were removed. ITS1, 5.8S, and ITS2 datasets were prepared with 182 representative species of *Hedysarum* plus nine genera, with 18 species, composed of *Caragana* Fabr., *Alhagi* Gagnebin, *Sulla* Medik., *Corethrodendron* Fisch. ex Basiner, *Onobrychis* Mill., *Ebenus* L., *Taverniera* DC., *Greuteria* Amirahm. & Kaz.Osaloo, and *Eversmannia* Bunge.

### 2.2 DNA extraction, amplification, and sequencing

DNA extraction and PCR reactions were performed based on Nafisi et al., [5]. AB101F and AB102R primers of Douzery et al., [67] were used for the amplification and sequencing of the nrDNA ITS region of the new taxa.

### 2.3 Molecular phylogenetic analysis

The full length of ITS1, ITS2, and ITS sequence-based phylogenetic analysis were performed using Maximum likelihood with the program RAxML-HPC2 on XSEDE [68]: Phylogenetic tree inference using maximum likelihood/rapid bootstrapping for 1000 replicates using GTR +G model for ITS2, and ITS and GTR +I+G for ITS1 regions run on XSEDE, after preliminary alignment in MAFFT [69] and manual adjustment. The models selected in the IQ-TREE web server [70] were based on the Akaike information criterion (AIC). The GC content of the ITS1, 5.8S and ITS2 sequences was determined using BioEdit v. 3.3.19 [71]. Also, DnaSP V. 6.12.03 [72] were used to implement diversity analyses and MEGA11 [73] for genetic distance analyses (Tables 1 and 2).

**Table 1:** The GC content of three ITS regions among clades.

	*H*. subsect. *crinifera*	*H*. subsect. *Multicaulia*	*H*. sect. *stracheya*	*H*. sect. Hedysarum	*Hedysarum*	CEGO clade
ITS1	55.71%	55.79%	54.25%	55.22%	55.51%	53.29%
5.8S	50.94%	51.55%	51.55%	51.57%	51.20%	51.31%
ITS2	53.21%	54.31%	53.12%	54.11%	53.55%	53.00%
ITS	53.29%	53.88%	52.97%	53.63%	53.42%	52.53%

**Table 2:** Between group mean genetic distances of ITS1 and ITS2 regions.

ITS1	CEGO	clade M	clade C	clade H
M	0.1744 ± 0.0300			
C	0.1589 ± 0.0250	0.0711 ± 0.0210		
H	0.1558 ± 0.0249	0.1295 ± 0.0323	0.0936 ± 0.0244	
S	0.1541 ± 0.0235	0.1291 ± 0.0327	0.0895 ± 0.0252	0.0957 ± 0.0249
M	0.1241 ± 0.0271			
C	0.0809 ± 0.0194	0.0770 ± 0.0214		
H	0.0736 ± 0.0167	0.1325 ± 0.0302	0.0956 ± 0.0238	
S	0.0472 ± 0.0116	0.1180 ± 0.0288	0.0672 ± 0.0193	0.0508 ± 0.0149

### 2.4 Inference of ITS1, 5.8S and ITS2 secondary structure

While the ITS1 and 5.8S boundaries were identified by software ITSx [74], ITS2 sequences were annotated from the alignment between the 5.8S and 28S gene proximal stem motifs using the new version of the web interface Internal Transcribed Spacer 2 Ribosomal RNA Database, ITS2-DB V [75], at http://its2.bioapps.biozentrum.uni-wuerzburg.de/. The complementary hybridization of both regions was observed using the ITS2-DB together with its ‘Annotate’ tool, which functions based on the hidden Markov models (HMMs). To predict the folding of the ITS2, RNAfold online tool, one of the core programs of the ViennaRNA package [76] using the dynamic programming algorithm originally proposed by Zuker and Stiegler [77], was queried for minimum free energy (MFE) secondary structures. Structural information was downloaded in Vienna and jpg formats for choosing a template. The predicted structure of *Hedysarum micropterum*QQ198828, due to fulfilling common core, possessing one of the lowest MFE in *Hedysarum*, and membership in the largest clade of the genus used as the template for folding the ITS2 sequences of the tribe via the custom modeling module of the ITS2-DB applying default parameters [78,79]. The inferred structures were examined for length, base composition, and GC content. The identified consensus secondary structures for the sections and subsections of *Hedysarum* were visualized via the web application from VARNA API doc V. 3–93 [80] and Inkscape 1.0.1. [81]. Subsequently, consensus secondary structures for the genus were obtained from 4SALE V. 1.7.1 [82]. The structures were conducted in the mfold web server [83] for the 5.8S and ITS1 regions for further analysis.

### 2.5 Alignment and sequence-structure analysis

Each ITS2 and ITS1 sequence dataset was simultaneously aligned with their secondary structures in 4SALE V. 1.7.1 using a clustalW binary file and was manually adjusted [82]. The maximum likelihood (ML) tree of seq- structs. was generated using package Phangorn 2.9.0 [84] as implemented in the statistical framework R. The available R script from the 4SALE homepage (http://4sale.bioapps.biozentrum.uniwuerzburg.de) was used including the model scripts. Bootstrap support values were estimated based on 1000 pseudo-replicates and the resulting tree was visualized with FigTree v.1.4.4 [85].

Due to a bug in cbcDetect of the CBCAnalyzer, the extracted CBC matrix from 4SALE was imported to the CBCAnalyzer to produce the CBC tree. Also, the CBCs were addressed from CBC matrix counts given in the consensus structure of multiple sequence-structure alignments in 4SALE.

## 3 Results

### 3.1 The length, GC content, diversity, and genetic distance analyses of regions

The length variation of the ITS1 region was moderately greater than ITS2 in the tribe, especially in the all genera except *Hedysarum*. The sequence of the ITS1 comprised 237−246 bases in *Hedysarum*, and 230−256 bases in the other Hedysaroid genera. ITS2 region comprised 221–227 bases in *Hedysarum*, and 220–228 bases in the other genera. And 5.8S sequences range from160 to 162 bases. *Onobrychis aucheri* and *Sulla aculeolata* in the ITS2 region, while, *Ebenus stellata* and *Caragana grandiflora* in the ITS1 region, were retrieved as the longest and shortest sequences.

ITS1 indicates more polymorphic sites, nucleotide diversity (Pi), parsimonious sites, the total number of mutations (Eta) and indel haplotypes, and approximately equivalent haplotype diversity than ITS2. On the other hand, ITS2 versus ITS1 revealed higher singleton nucleotides, haplotypes, indel sites, and indel haplotype diversity (S12 Table).

As expected, 5.8S and ITS1 regions were found with the lowest and the highest GC content for *Hedysarum*, respectively (Table 3). Subsequently, Clades M and CEGO had the highest and lowest GC content in ITS1 and ITS2, respectively. In the case of the 5.8S region, both clades M and S indicated the highest values of GC content and revealed the lowest value in clade C. The GC content information of clades is presented in Table 3.

**Table 3:** Within group mean distances.

	GECO	clade M	clade C	clade H	clade S	Total
ITS1	0.0981 ± 0.0161	0.0071 ± 0.004	0.0125 ± 0.0045	0.242 ± 0.0055	0.0252 ± 0.0062	0.08 ± 0.01
ITS2	0.0478 ± 0.0105	0.0029 ± 0.0025	0.0095 ± 0.0037	0.0212 ± 0.0053	0.0114 ± 0.0041	0.07 ± 0.01

### 3.2 Sequence analysis and reconstruction of phylogenetic trees

After mining the close genera in GenBank, for the determined boundaries of ITS1 and 5.8S regions and then annotating the ITS2 region in ITS2DB, the boundary of 5.8S−ITS2 (5’ “CATAT” 3’) was verified three nucleotides following the depicted point in Genbank. Consequently, three nucleotides of the start point of 5.8S were pruned, after annotation by the software ITSx.

#### 3.2.1 ITS1 sequence and sequence-structure based ML trees

**Fig 1:**
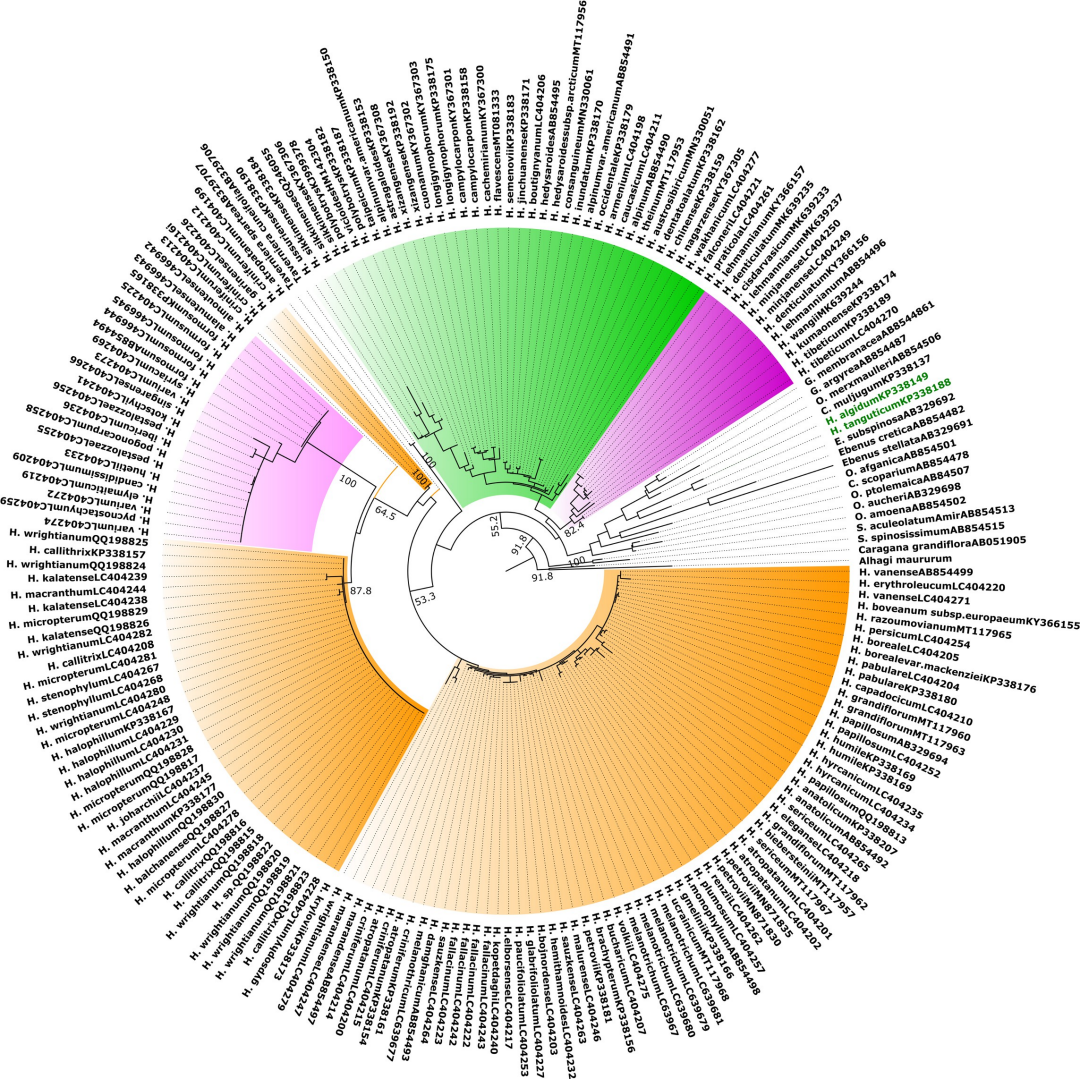
ITS1 sequence-structure Maximum-Likelihood tree calculated with R including a representative subset of 204 sequence-structure pairs from *Hedysarum* and its affiliated genera. Bootstrap support values from 1000 pseudo-replicates greater than 50% mapped at the internodes.

The ITS1 seq. and seq-struct-based phylogenetic trees displayed two inconsistent topologies. In the sequence-based tree, *Ebenus* placed in a close relationship with a lineage (BS = 54) comprising clade H (BS = 91) and *Taverniera* (BS = 100), with low support value (BS = 39). And, the CEGO clade (BS = 95) placed near (BS = 21) a weakly supported node (BS = 58) composed of clades S (BS = 96), C (BS = 95) and M (BS = 100). In the sequence-structure tree (Fig 1), *Ebenus* was placed inside the CEGO clade in a close relationship with clades H and S (BS = 82.4). whereas, *Taverniera* united with clades M and C. However, these nodes didn’t improve with reasonable support values. Meanwhile, unexpectedly, this tree couldn’t diverge a monophyletic clade C.

Concentrating on species of west Asia, species groups have been monitored through the clades C and M in all trees. Whereas, in ITS1 seq. tree, H. wrightianum group (A), H. criniferum (B) (+ *H*. *marandense*), H. kopetdaghi (D), and H. monophyllum (E) groups were distinguished. Only, A, D, and E groups have been retrieved in ITS1 seq-struct tree. Also, ITS1 trees have been more successful in resolving species of clade M.

#### 3.2.2 ITS2 sequence and sequence-structure based ML trees

**Fig 2:**
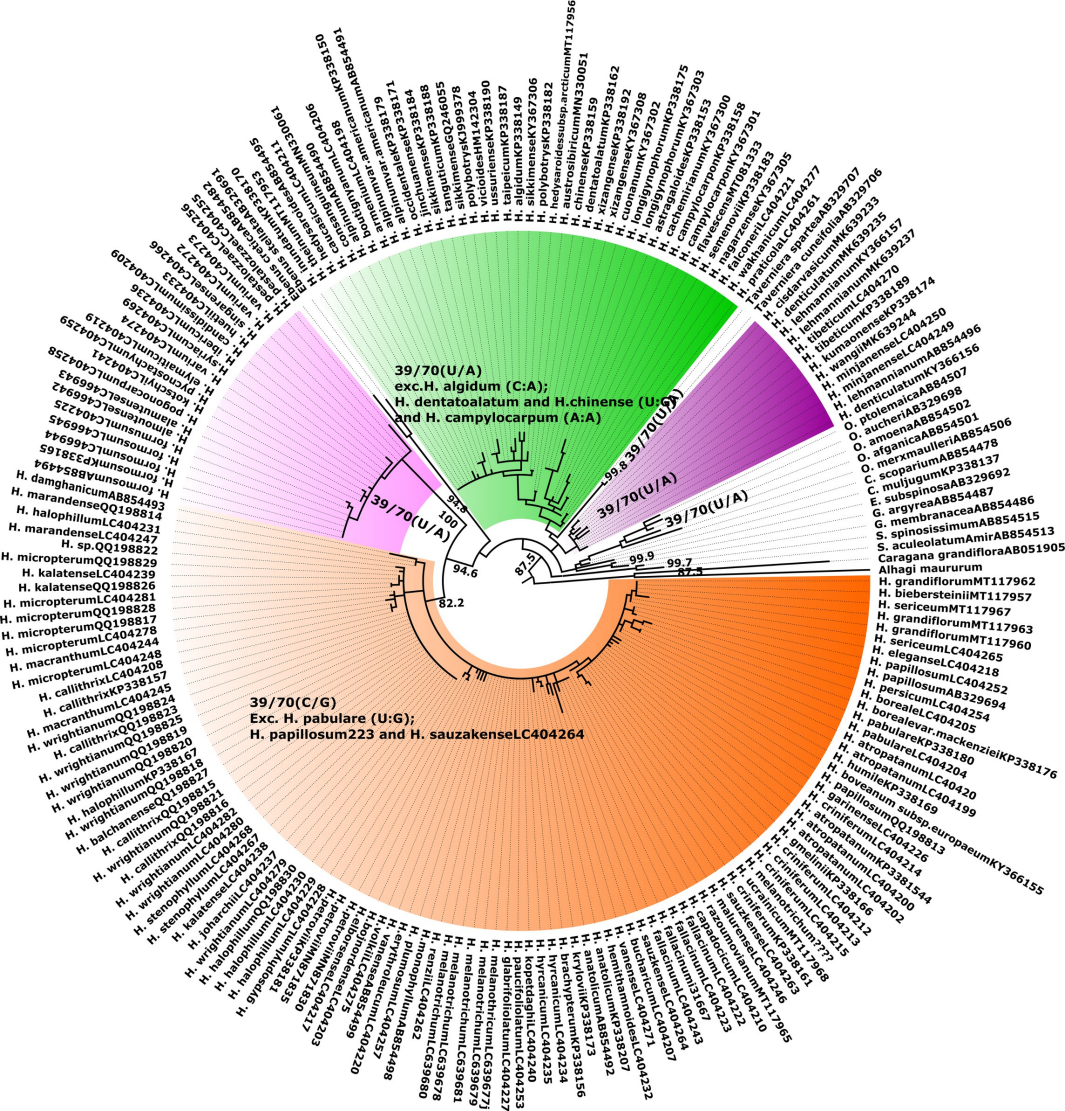
ITS2 sequence-structure Maximum-Likelihood tree calculated with R including a representative subset of 204 sequence-structure pairs from *Hedysarum* and its affiliated genera. Bootstrap support values from 1000 pseudo-replicates greater than 50% mapped at the internodes. The blue blocks represent conserved motifs.

The ML tree of both sequence and sequence-structure of the ITS2 region generated almost the same tree topology. In the synchronized sequence-structure tree (Fig 2), a lineage (BS = 48.5) is comprised of three clades in polytomy. First the CEGO clade (BS = 29.4), followed by a clade (BS = 23.6) consisting of clade S (BS = 65.5) in close relationship with, *Taverniera* and clade H (BS = 31) (BS = 10). The final clade (BS = 95) comprised of clades C (BS = 82.2) and M (BS = 100), both in close relationship with *Ebenus*. In sequence only tree, *Ebenus* set inside of CEGO clade, and *Taverniera* showed a close relationship with clade S with low support value. Also, the seq-struct ML tree improved the support value of the deep node of diverging *Sulla*. In terms of species groups, in additionto reconstructing H. fallacinum (C) and H. wrightianum (A) groups in ITS2 seq. tree, in the ITS2 seq-struct tree, *H*. *sericeum* group (consisting of *H*. *sericeum*, *H*. *elegance*, *H*. *grandiflorum*, and *H*. *biebersteinii*) was determined, too.

### 3.3 ITS1 alignment and secondary structure

**Fig 3:**
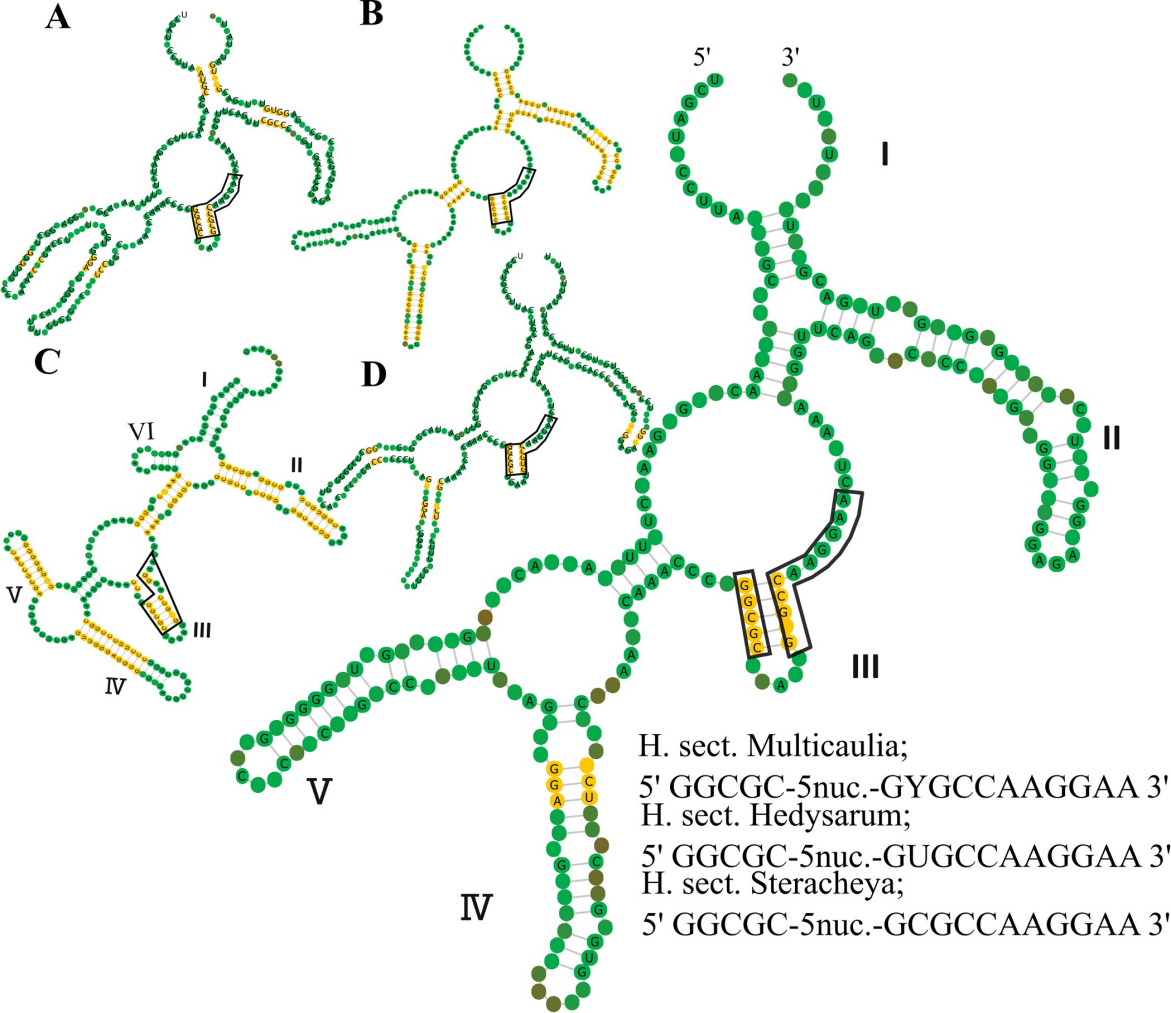
Olsen layout of ITS1 100% conserved consensus secondary structure of A. *H*. sect. *Multicaulia* subsect. *Crinifera* (clade C). B. *H*. sect. *stracheya* (clade S). C. *H*. sect. *Multicaulia* subsect. *Multicaulia* (clade M) and d. *H*. sect. *Hedysarum* (clade H). The blocks represent a conserved motif.

There were 44.4% universally conserved nucleotides among the 199 studied taxa. The ITS1 consensus secondary structures of *Hedysarum*, according to optimal minimum free energy (MFE), are illustrated in Fig 3. The secondary structures of the CEGO clade have been choosing from five optimal and suboptimal structures.

ITS1 secondary structure of clade H consisted of one large (second or third loop) plus two small central loops classified into two types. First loop with two, the second with zero, and the last with three helices, or first loop with two, second with one, and the last with two helices. The length variations of helices through clades in ITS1, and ITS2 regions are illustrated in Table 4. Furthermore, clade S indicated one structure, comprising a large plus two small central loops, the first loop with two, the second with one, and the last with two helices. Additionally, in the clade M, *H*. *formosum*, *H*. *alamutense*, and *H*. *syriacum* constructed structures with three central loops, first with two helices, second with one, and third with two helices. Other species with one additional helix on the first loop. Further, were observed two central loops with six helices in *H*. *varium*LC404273. Moreover, clade C incorporates two structure types, first with three central loops consisting of one large plus two small, the first loop with two, second with one, and the last with two helices, the next with two central loops, first with two helices and second with three helices. The delta G required for the formation of the secondary structures of CEGO, H, M, S and C clades were on average -94.08, -92.58, -92.82 95.40 and -95.64 kcal/mol, respectively, composed to -94.10 kcal/mol for ITS1 region of *Hedysarum*.

**Table 4:** Length of Helices of ITS1, ITS2 in clades.

	Helix 1; I		Helix 2; II			Helix 3; III		Helix 4; Ⅳ		Helix5	Helix6	
clade H	22−31	40		42−53	37−39	15−23	83−86	37−38	14	25−39		-
clade M	33	41		43	39	23	85	38 (-37)	14−18	21		14
clade C	30	40		40,50,52	38−40	15−23	85−87	33,40	14	35−42		-
Clade S	22−31	40		52	39−40	15	85	39,44	14	37−43		-

In the ITS1 consensus secondary structure model of *Hedysarum*, eight base pair positions were 100% conserved in all taxa, including 5 in Helix 3 and 3 in Helix 4. The abundance of 100% conserved base pairs in the consensus ITS1 secondary structure models of detached datasets of main clades represents below. Fifty-four base pair positions in the model of clade S were 100% conserved, as 5 in Helix 1, 18 in Helix 2, 5 in Helix 3, 12 in Helix 4, and 14 base pairs in the skeleton. The consensus secondary structure of clade S with 54 bp 100% conserved position, followed by clade M with 51 bp, clade H with 20 bp and clade C with 19 bp.

Species corresponding to each genus in the CEGO clade indicated variable structures. *Onobrychis merxmuelleri* and *Greuteria* indicated a specific structure with two central loops and six helices, one loop with two and the other with four helices, versus other *Onobrychis* species and other genera in the clade, with three central loops and five helices, as two external loops each with two and the middle one with one helix. Only, *Ebenus cretica* indicated three helices in the first loop and *Sulla* retrieved two central loops and five helices, distinct from the other genera. There were 2–11 non-canonical G-U base pairings in *Hedysarum* consensus structures. Clade M possessed the highest number of G-U base pairings.

The screening CBC table of ITS1 region shows CBCs only among *O*. *ptolemaica*, *O*. *amoena* (sect. *Hymenobrychis*), *O*. *aucheri* (Sect. *Heliobrychis*) and *O*. *afghanica* (Sect. *Dendrobrychis;* CEGO clade), and *Hedysarum*. *Onobrychis aucheri* made CBCs with the other three genera in the clade and *O*. *amoena* and *O*. *ptolemaica* only with *Greuteria*. We did not detect any CBCs between the other three genera. Also, *Taverniera* and *Ebenus cretica* constructed CBCs with all genera [except *Taverniera* and most of clade S]. Within *Hedysarum*, the CBCs are perceived between clade M and H, between S and H, and between *H*. *formosum* and *H*. *alamutense* and *Hedysarum* main clades.

#### 3.3.1 ITS1 region motifs

Angiosperm universal core motif [4] was detected as 5’ GGCGC-(4 n)-GYGCCAAGGAA 3’ in ITS1 regions through all datasets. 5’ GGCGC and GYGCC are restricted to the stem region of Helix III. Therefore, this highly conserved motif forms a hairpin structure, which means these ITS alleles are functional. 5’ UCAG vs. 3’ AGUU at the base of Helix 2 is taxonomically conserved. *Corethrodendron*, *Greuteria*, *Eversmannia*, and clade H, indicated 5’ GUGCCAAGGAA 3’; *Alhagi*, *sulla*, *Ebenus*, *Caragana*, *Taverniera*, clade S and C, possess 5’ GCGCCAAGGAA 3’; clade M and *Onobrychis* indicated 5’ GYGCCAAGGAA 3’. *Hedysarum formosum* and *H*. *alamutense* are the only species in clade M with “U” in the second nucleotide.

### 3.4 5.8S structure and motifs

Similar to the other prediction tools, most of the predicted secondary structures of the 5.8S gene didn’t meet the known structure of eukaryotes, therefore the tenth suboptimal minimum free energy structures were inferred from mfold. *Alhagi* (with two helices), and members of the CEGO clade (literally with one helix) couldn’t retrieve the inferred structure. All *Hedysarum* species shared a common structure with one central loop and four conserved helices except *H*. *garinense* with two plus one branched helix. The structure showed relatively uniform stability, ranging from -45.00 to -49.50 kcal/mol for the tribe and -47.10 to -47.60 kcal/mol in the case of *Hedysarum*. *Hedysarum citrinum* and *H*. *neglectum* were neglected from the dataset because of their changes in motifs. In the 5.8S consensus secondary structure model of *Hedysarum*, 32 bp were 100% conserved in all taxa, including 25 in Helix 1, 3 in Helix 2, and 4 in Helix 3. The proportions of GC pairs ranged as Helix 1 55.5%; Helix 2 33.3%; Helix 3 75%; and Helix 4 60%. And proportions of G-U pairs in the helices are as bellow, Helix 1%11; Helix 2%0, Helix 3 25%; and Helix4 40%.

**Fig 4:**
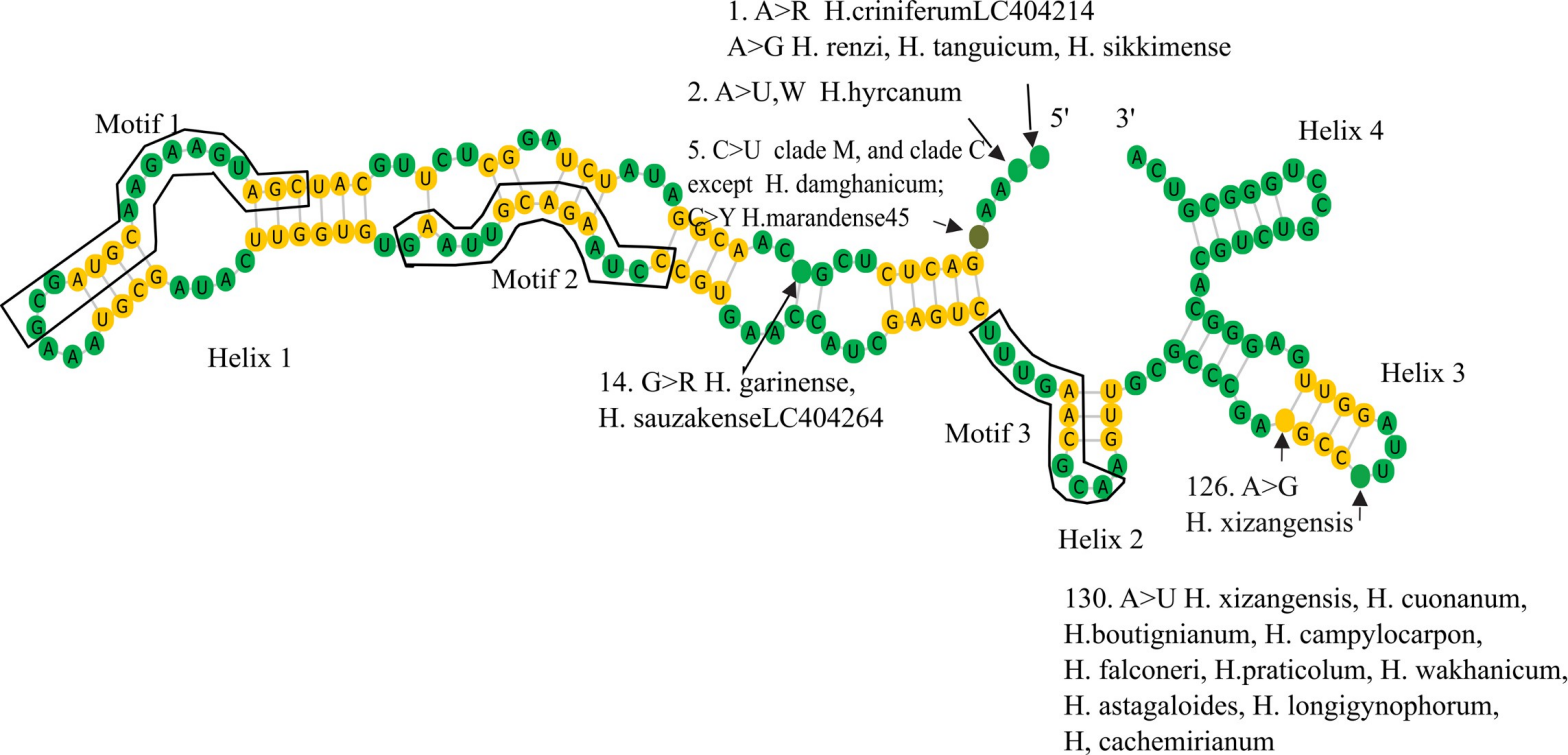
Olsen layout of 5.8S region 100% conserved consensus secondary structure.

The presence of three conserved angiosperms 5.8S motifs viz. Motif I: 5′-CGAUGAAGAACGUAGC-3′ [35]; Motif II: 5′ GAAUUGCAGAAUCC-3′ [35]; Motif III: (5′-UUUGAACGCA-3′) [86] in the 5.8S region of all the studied taxa was also observed), indicating no pseudogenes in the data set. Motif 2 is situated 25 bases downstream from the conserved ‘AAGAA’ sequence that takes part in the formation of a loop structure in Helix I in all flowering plants [87]. Base substitutions through the *Hedysarum* species are illustrated in Fig 4, and cataloged in S11 Table.

### 3.5 ITS2 secondary structure

ITS2 region comprised 53.3% universally conserved nucleotides among the 199 studied taxa. Preceding the structure prediction in RNAfold online tool, the 5’ and 3’ end of the ITS2 region were added with 25 bp of the 5.8S and 28S rRNA sequences, respectively. The added sequences aid in canonical base pairing and folding of the ITS2 region [79,88,89] and the high stability of structures. As, the delta G required for the formation of secondary structures of the ITS2 region of *Hedysarum* was, on average, -103.25 kcal/mol. The highest and lowest delta G belongs to clade H with -104.97 and clade S with -101.79.

**Fig 5:**
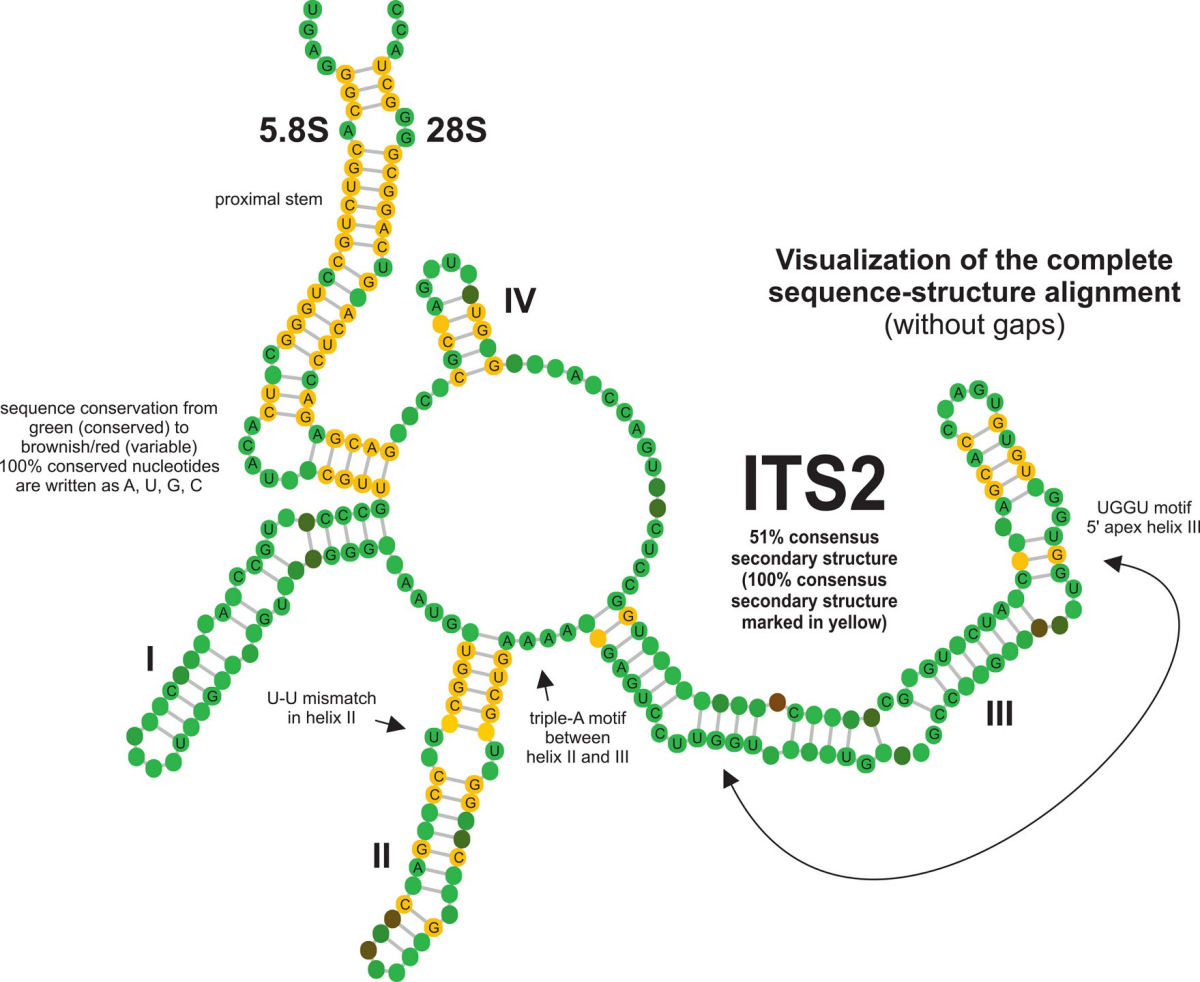
ITS2 consensus secondary structure scheme, with 100% conserved positions through the tribe.

**Fig 6:**
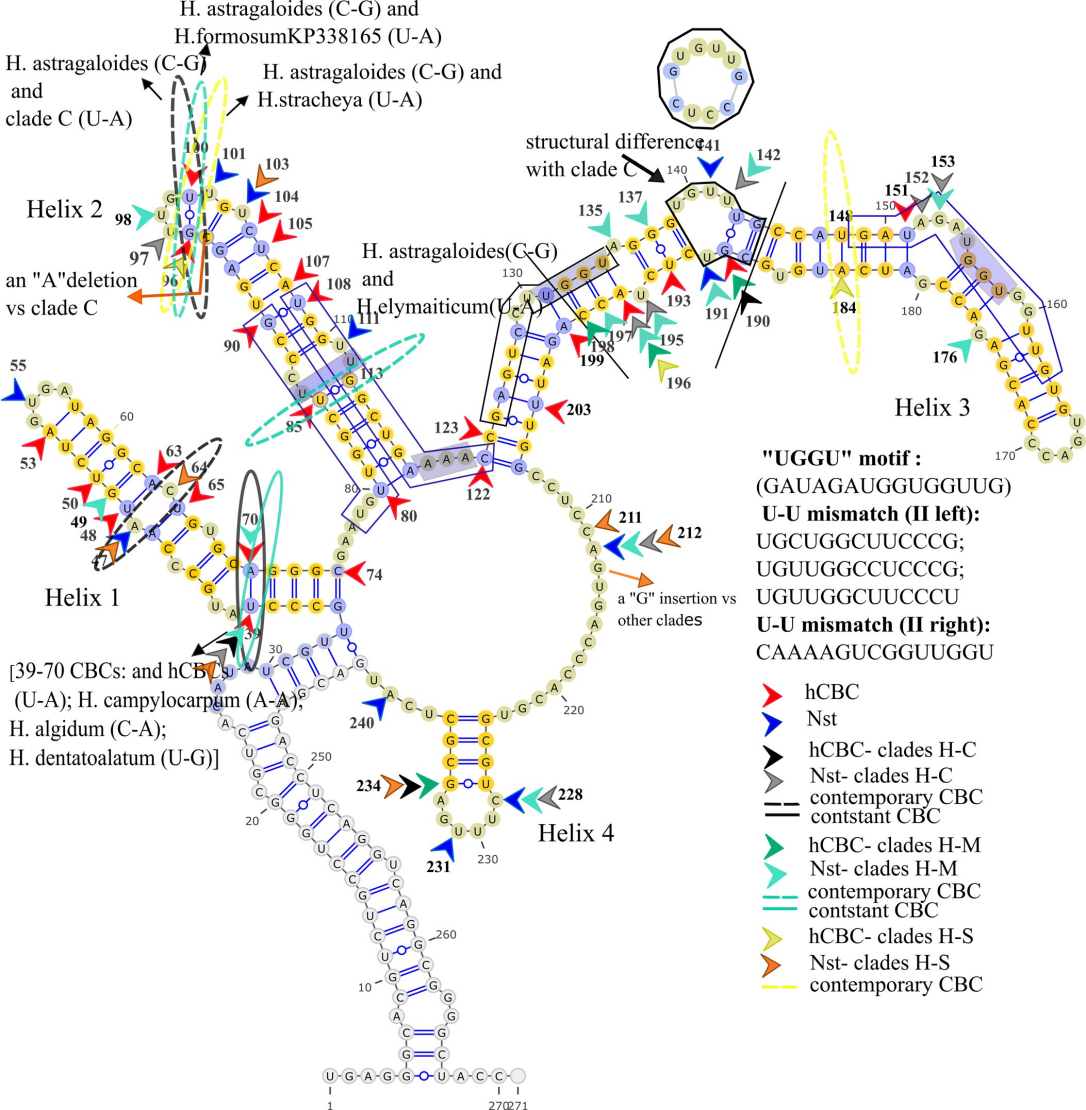
ITS2 Consensus secondary structure of *H*. sect. *Hedysarum* (clade H). Yellow flashes 100% conserved regions in the section.

**Fig 7:**
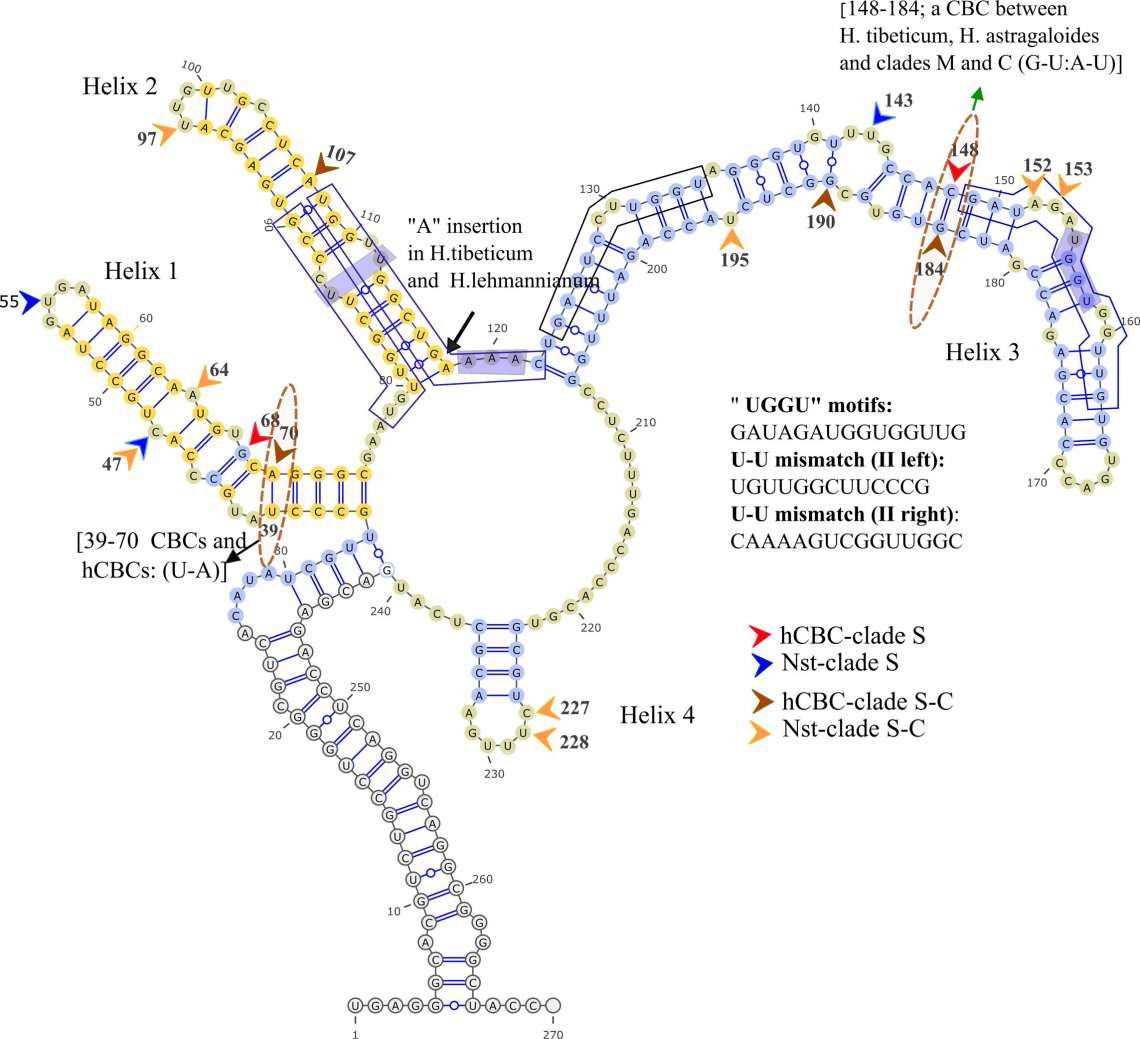
ITS2 Consensus secondary structure of *H*. sect. *Stracheya* (clade S). Yellow flashes 100% conserved regions in the section.

**Fig 8:**
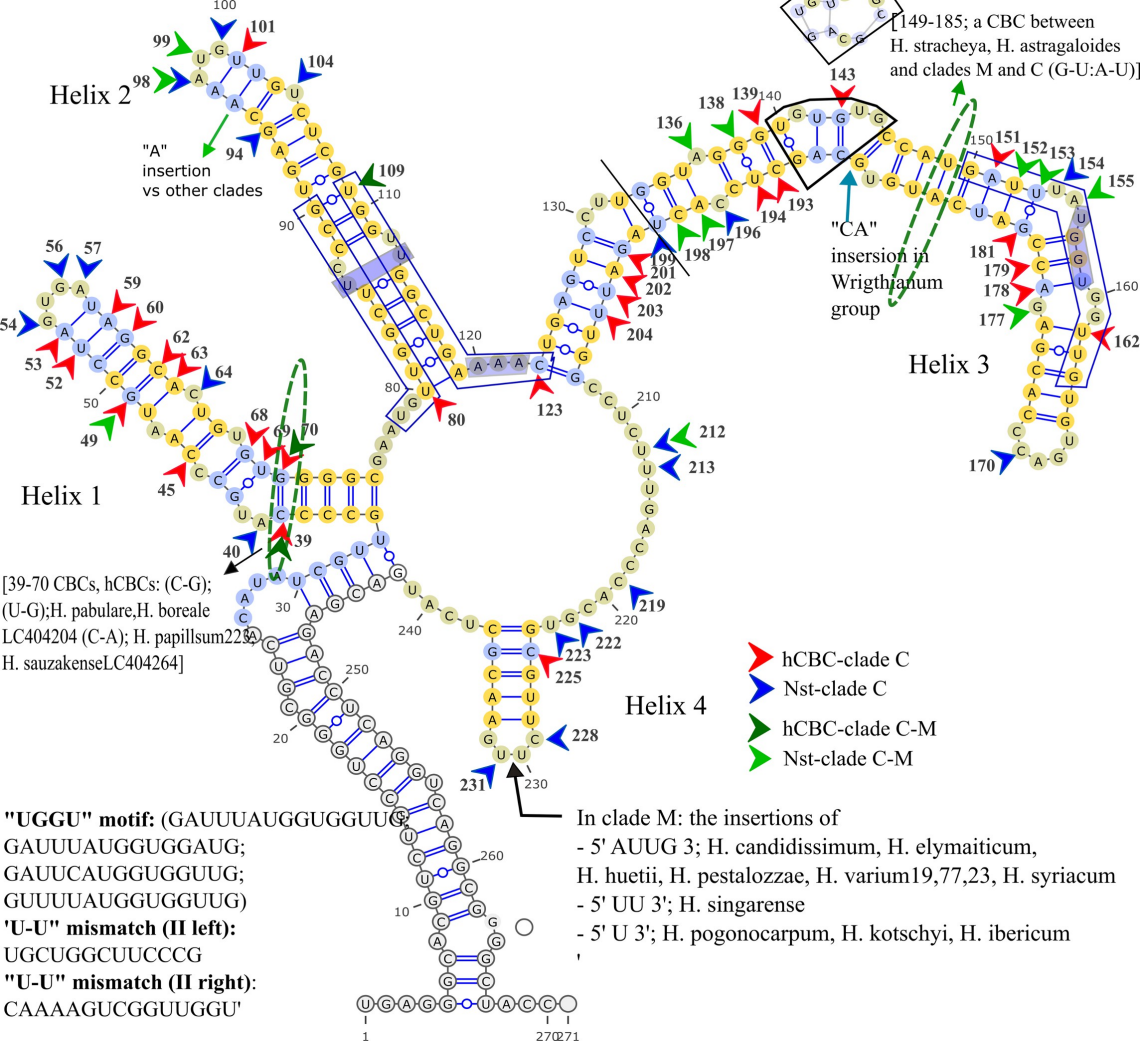
ITS2 Consensus secondary structure of *H*. sect. *Multicaulia* Subsect. *Crinifera* (clade C). Yellow flashes 100% conserved regions in the section.

**Fig 9:**
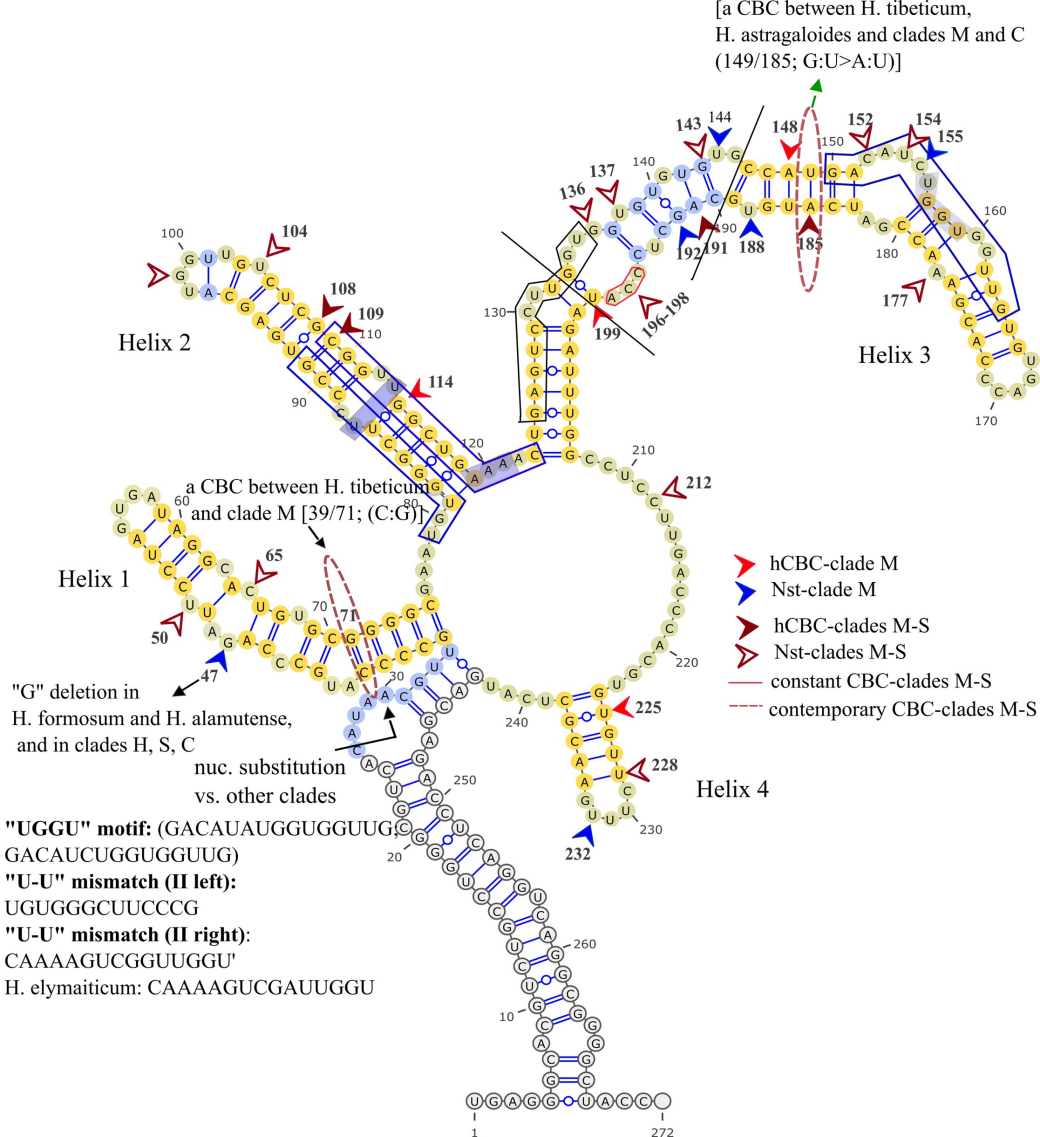
ITS2 Consensus secondary structure of *H*. sect. *Multicaulia* Subsect. *Multicaulia* (clade M). Yellow flashes 100% conserved regions in the section.

The secondary structure through the tribe presents four helices radiating from a central loop beside the proximal stem. To set against, a consensus structure for *Hedysarum*, with 100% conserved base pairs through the tribe (Fig 5), and a distinctive consensus secondary structure for each section and subsection of *Hedysarum* (Figs 6–9).

As sequence variability of helices, Helix I (0.75%) and Ⅲ (0.61%) of ITS2 consensus secondary structure of *Hedysarum* possess the highest proportion of variables (variant sites/ total sites), and Helix Ⅳ (0.5) the lowest proportion. The examined number of transition to transversion mutations in the ITS2 region in the clades as, Clade H (ti: tv, 23:11), clade C (28:14), clade M (7:1), clade S (1:3) reaches 59:29 in the genus.

The helices lengths of secondary structures of each clade are represented in Table 4. In the ITS2 consensus secondary structure of the genus 17 bp (of 68; 25%) 100% conserved pairs have been detected (Fig 5); nine bp (of 16; Helix II), five bp (of 32; Helix III), and three bp (of five; Helix Ⅳ). Also, considering the foot of helices, four conserved nucleotides have been found on the 5’ side and three bases on the 3’ side of Helix I, four bases on the 5’ side and five on the 3’ side of Helix II, and 11 bases on the 5’ side of Helix III. The high degree of conservation in the structure allowed the unambiguous alignment of most of the ITS2 positions, and the generation of a consensus secondary structure model of the ITS2 in the tribe [18]. In terms of G—U pairing, Helix Ⅱ of ITS2 consensus secondary structure with 25% (G-U bp/ total pairs), and helix Ⅲ with 20% show the highest proportions and helix Ⅰ with 2.1% the lowest rate.

The stem consisting of 5.8S and 28S reunion shows the same structure of stacking and loops through the tribe, except for “T>A” in 30^th^ nuc. in a bulge loop, a specific character for clade M. In this subsection, *H*. *alamutense* shows a diverse nuc. in position 29 (A> U, N), too. Moreover, *Alhagi* indicated mutations in 21 (C>U), 27 (A> G), and 290 (C>U) positions, and *Corethrodendron multijugum* in 290 (C>U), too.

#### 3.5.1 ITS2 region motifs

Sequence variations, such as “UGGGU”, “UGG”, or “GGU” (Helix III, 5’ side) have been described in addition to the existence of a U-U mismatch (Helix II, left and right) plus AAA between helices II and III which is conserved in the vast majority of eukaryotes [17,62]. The variation of the UGGU motif region is perceived in *Hedysarum* clades (Figs 5–9), *Greuteria* (GAUAGAUGGUGGCUG), and *Alhagi maurorum* (GAUCGACGGUGGUUG). Also, the U-U mismatch (II, left) motif region variations in clades H and C were marked (Figs 6 and 8). Moreover, the U-U mismatch (II, right) motif region variation in *Caragana grandiflora* (UAAAAGUUGGUUGGU), clade S and *H*. *elymaiticum* were detected (Fig 7).

### 3.6 ITS2 CBC, hCBC and Nst analyses of clades

We have found six CBCs in the ITS2 consensus secondary structure of the tribe: A CBC at positions 148/186 (not-aligned, Helix III) of *Alhagi* (U:G) versus other genera (A:U), and *H*. *tibeticum* versus all taxa (except clade S and *Caragana*); at 39/70 (not-aligned, Helix I), between CEGO, clades S and H (U:A) [except, *H*. *campylocarpon* (A/A), *H*. *algidum*(C/A); *H*. *chinense*, *H*. *dentatoalatum* (U/G)], and *Taverniera* (C:G), clades M, and C (C:G) [except, *H*. *pabulare* (U:G), *H*. *papillosum*QQ198813 and *H*. *sauzkense*LC404264 (C:A), hCBCs]; at 47/64 (not-aligned, Helix I, (G:C > A:U or A:Y) between *H*. *longigynophorum* (clade H), and *H*. *macranthum* and *H*. *micranthum* (clade C); at 85/113 (not-aligned, Helix II; U:A), *H*. *elymaiticum* (clade M) versus *H*. *longigynophorum*, *H*. *astragaloides*, *H*. *praticola*, *H*. *wakhanicum*, *H*. *falconeri*, *H*. *xizangensis*, *H*. *cachemirianum*, and *H*. *flavescens* (clade H, C:G); at position 97/101 (not-aligned, Helix II, G:C) exclusively *H*. *astragaloides* versus *H*. *praticola*, *H*. *wakhanicum*, *H*. *Falconeri* (A:U, clade H), and clades S, and C (except *H*. *anatolicum*, *H*. *vanense* LC404271), clade M (except *H*. *singarense*), CEGO clade (except *Greuteria*), *Ebenus stellata*, *Sulla*, and *Caragana*. In other word, a CBC exhibited between *Taverniera* and clades S and H. Moreover, no CBC is detected between clades C and M or between *Ebenus* and other clades.

In inter-sectional or inter-sub-sectional level of *Hedysarum* ten events of hCBC and 37 Nst happened in ITS2 spacer. Clades H-M with six hCBCs, and 15 Nsts and S-H clades with two hCBCs and 11 Nsts showed the highest and lowest changes between clades. In the intra-sectional or sub-sectional level, clade C with 10 hCBCs plus 29 Nsts and clade H with 13 hCBCs plus 26 Nsts were recorded as the most variable clades, and clade M with five hCBCs, plus three Nsts as the least interspecific variable clade. On the other hand, two CBCs, seven hCBCs, and 23 Nsts in Helix Ⅰ; two CBCs, and six hCBCs, and 11 Nsts in Helix Ⅱ; one CBC and 14 hCBCs, and 18 Nsts in Helix Ⅲ; and two hCBCs, and five Nsts in Helix Ⅳ, were monitored. In this regard, Helix Ⅲ is specified as the most variable, and Helix Ⅳ as the most stable. Parallel with the highest number of G-U base pairings in Helix Ⅲ. Entire nucleotide changes of intra and inter-sections and subsections of *Hedysarum*, in the ITS2 region, were cataloged in S1–S11 Tables and the cites were elucidated in Figs 6–9. Our study also highlights that the helices of the secondary structures of both spacers have more variations than loops. Considering 89 Nsts and 32 hCBCs in *Hedysarum* which nearly half of Nsts are concentrated on the helix portions of spacers than loops, the faster evolution of loops than helices, assumed from more GC- rich content by Escobar et al., [90], is not conceivable.

### 3.7 ITS1 and ITS2 secondary structure features of species

The detected hCBCs and Nsts in species and species groups assigned by Nafisi et al., [5] are considered here. An hCBC in Helix Ⅰ of ITS2 structure of *H*. *armenium* versus *H*. *caucasicum* (35/74 not-aligned, C>U, clade H), and an Nst (215, U>K) in Helix 2 of ITS1 support two species. Whereas *H*. *melanothricum*, three samples of *H*. *criniferum*, and *H*. *atropatanum* possess a distinct ITS1 secondary structure versus three separate accessions of *H*. *criniferum*, two first species rendered an Nst in Helix 3 of ITS1, and two hCBCs in Helix Ⅰ and Ⅲ of ITS2 secondary structures. *H*. *damghanicum* Rech.f. separate from the group (B) members with three hCBCs in Helix Ⅲ and Ⅰ plus two Nsts in Helix Ⅰ of ITS2.

While, *H*. *sauzakense* Rech.f. group (C) versus the *H*. *kopetdaghi* Boriss. group (D) didn’t specify any informative changes in the structure of the ITS1, signified with two Nsts located in Helix Ⅰ of ITS2. Indeed, between two groups in ITS1 dataset, there is an informative nucleotide site (149 of aligned, G>C) but a rearrangement of nucleotides at the tip of Helix 4 made any sense of CBC or hCBC. On the other hand, *H*. *bojnordense* shared the same set of nucleotides with *H*. *kopetdaghi*KP338172 and commonly shared an Nsts in helix 3 of ITS2 structure in contrast with the group (D), while in ITS1 *H*. *bojnordense* shared identical nucleotides with *H*. *kopetdaghi*LC404240 and differ with KP338172 in an Nst in Helix 1. Moreover, in this group, two Nsts have been found in helices 4 and 5 between *H*. *elbursense and H*. *hyrcanum* in the ITS1 region. As mentioned in Nafisi et al. [5], there is only one decrepit type specimen of *H*. *elbursense*. The equal locality of this type species and some morphologic species led us to synonymize the two species. *Hedysarum fallacinum* (including *H*. *longipedunculatum*; group C) shared the same nucs. and is designated with an Nst at Helix Ⅰ of ITS2 and with an insertion at the 3’ ending of the ITS1 region. *Hedysarum persicum* retrieved with one Nst at Helix Ⅲ of ITS2 versus *H*. *papillosum* Boiss. (W >U), with no identifier at the ITS1 region. Also, *H*. *pabulare* distinguishes from close species *H*. *boreale* with two hCBC in Helix Ⅲ and Ⅰ of ITS2, two Nsts in Helix Ⅲ and Ⅳ and one Nst in Helix Ⅳ of the ITS1 region. *Hedysarum wrightianum* (A) and *H*. *criniferum* (B) groups were delimited with two hCBCs in Helices Ⅰ and Ⅲ, plus two Nsts located in Helix Ⅲ and central loop. The given hCBC in Helix Ⅰ of ITS2 is detected in a clade of three specimens of *H*. *macranthum* and *H*. *micropterum* (A). Nevertheless, we found *H*. *kalatense*, *H*. *johartchii*, and *H*. *balchanense* with identical ITS2 regions located in an immense polytomy, composed of nine species.

In clade M, each *H*. *elymaiticum* and H. formosum group (*H*. *formosum* and *H*. *alamutense*) are specified with a distinct hCBC in ITS2, the other species share four hCBCs. In addition, the H. formosum group specifies an Nst in Helix Ⅲ and a nucleotide deletion in Helix Ⅰ. Also, in the ITS1 region, a CBC was perceived between this group and clade C.

Consequently, however, hCBCs of ITS2 were mainly successful in identifying species groups but also improved the relationships between a few close species like *H*. *melanothricum− H*. *criniferum*, *H*. *damghanicum− H*. *criniferum*, *H*. *plabulare− H*. *Boreale*, and *H*. *armenium− H*. *caucasicum*. It is noteworthy that we encountered many Nst cases occurred in one species representatives.

## 4 Discussion

### 4.1 Phylogenetic inferences of ITS, ITS1 and ITS2

This is the first study to systematically evaluate the predicted rRNA secondary structures of the tribe Hedysareae and assess their phylogenetic implications. Whereas the ITS1 seq. tree corresponds to the whole ITS seq. tree in the topology of main clades and virtually the reconstruction of the species groups, ITS1 seq-struct ML tree, retrieved the consensus tree topology of five nuclear regions of Liu et al., [6], with lower support values; clades S and H in the close relationship with CEGO clade, and *Taverniera* with the Sartoria clade (including C and M clades). Whereas, in all seq. trees *Taverniera* is being joined with clade H or S clade. In both sequence and seq-struct ITS2 trees, the relationships between the main clades stayed unresolved, howbeit of clade S correctly placed near clade H. Since, none of the synchronous trees could improve the bootstrap values; therefore, exclusive ITS, ITS1, and ITS2 do not seem to be suitable markers for distinguishing the relationships of clades and species groups. But the synchronized seq-struct of ITS1 is recommended for the reconstruction of main clades.

### 4.2 Length, GC content, diversity and genetic distance analyses of the regions

All three regions lie within the upper intermediate range of sequences reported from previous plant studies of rDNA [31,62,87]. The 5.8S region displayed a nearly uniform length throughout the tribe. The ITS1 and ITS2 sequences showed nine and six bases variations in *Hedysarum*, and 26 and eight bases in the tribe. Furthermore, ITS1 shows the greatest length of three regions.

The average GC content of the three regions of ITS in *Hedysarum* was similar to values observed in other plants [87]. The observed similar GC content of ITS1 and ITS2 markers in this case study implies that they are authentic sequences under functional and selective constraints and not pseudogenes, based on former reports [7,86,91]. On the other hand, the GC content of the CEGO clade is similar to clade S in both regions which is in parallel with the genetic distance results (Table 1).

The more variable and rapid evolution of ITS1 than ITS2 was confirmed formerly in plants and fungi [22,57,58,66,92]. The current analyses also recovered most of the diversity indices of ITS1 more variable than ITS2. Also, ITS1 demonstrated a higher mean distance between clades. Consequently, this region retrieves better ability in phylogenetic implications. In the ITS1 region, clades C-M, and afterward clades S-C indicated the lowest mean genetic distances. In comparison, ITS2 shows the lowest genetic distances between CEGO-S and S-H, in order. In both regions, the CEGO shows the lowest distance with S and H clades (Table 1), in respect. The results except for the closeness of clades C and S are consistence with the nuclear tree topology of Liu et al. [6] and ITS1 synchronous seq-struct tree. On the other hand, Clade M and clade H show the lowest and highest within-group mean distance in both regions (Table 2), which confirms the reported ITS2 hCBC and Nst events from clades (clades C and H collectively were the most variable in this analysis).

### 4.3 ITS1, 5.8S and ITS2 consensus secondary structures

The ITS1 and ITS2 regions are already well known to play important roles in the rRNA maturation process [4,13,22,56,61,63,91,93,94], apparently requiring secondary structure, despite dramatic nucleotide sequence variation. Also, the 5.8S rRNA plays a critical role in ribosome movement and protein translation and therefore, displays a high degree of pan-eukaryotic conservation [29,95].

Uniform length and very low levels of sequence variation in the 5.8S gene, along with no substitutions in the ITS1 motif of Liu and Schardl [4] or the three highly conserved 5.8S gene motifs [62,86,96] or key conserved structural motifs of ITS2, in the vast majority of eukaryotes [4,17,97], amongst all samples is a good indicator that potentially functional ITS sequences and thus valid gene copies have been generated [86,97,98]. Therefore, invalid ITS sequences that would otherwise negatively affect phylogenetic reconstruction were removed from the data set.

The 5.8S rRNA in *Hedysarum* shows 86.3% to 94% homology with the corresponding gene in *Canella winterana* [87]. Since most of the structures in mfold (and other tools like RNAstructure and RNAfold) constructed a stem in the 5’ site instead of a loop, we considered the tenth suboptimal structure of the tribe sequences approving the structures of eukaryotes, a structure with one central loop and four conserved helices [29,87]. The structures of the 5.8S region of *Hedysarum* indicate uniform stability based on thermodynamic energy values. and more negative ∆G representing more stable structures to produce. Disregarding three autapomorphic sites, the three informative substitutions (t_i_: t_v_; 4:2) were detected in this region. Two nucs. substitution in the 5’ side of the loop: in the first site identify *H*. *renzi*, *H*. *criniferu*mLC404214, *H*. *tanguicum* (clade C), and *H*. *sikkimense* (clade H), and in the fifth site characterizes clades C-M (with two exceptions). Moreover, the substitution in point 130 (aligned, Helix 3) developed in ten species of *H*. sect. *Hedysarum*.

The Hedysareae ITS2 secondary structure is comparable to those of other eukaryotes [17,49,61]. The four helices radiating from a large central loop proceed through the tribe by force homology modeling of ITS2DB and RNAfold tool. We examined the optimal secondary structures of *Hedysarum* ITS1 based on the optimum minimum free energy and acquired five distinct structures for *Hedysarum* with uniform thermodynamic energy lower than that of ITS2. The structure predictions were proceeded by adding A- rich 5’ end of 5.8S to and or eliminating the 3’ wobble end of ITS1 but a central loop with radiating helices did not obtain. Therefore, having fewer functional limitations, the rDNA ITS1 region, typically shows more variability both in sequence and structural level, in terms of helices and loops number, in inter or intra−sections levels of *Hedysarum*. Whereas, ITS2 structures gained from force homology modeling of ITS2DB show a variation of 2–10 nt in the length of helices and the number of bulges and internal loops. Due to these fluctuations in the tribe, we used a pattern to model all taxa accordingly. The present structures verify the conservation of basal pairings of helices I and II of ITS2 and serve as a scaffold for shaping the structure [99]. The fluctuations in the ITS2 motif regions appeared as an identifier of sections and subsections. Clade C and H possess the most variable UGGU and U-U mismatch motif regions (II, left). Clade S was retrieved as the most rigid in both regions, yet shows fluctuation in the U-U mismatch (II, right). ITS2 region has a higher t_i_: t_v_ bias than ITS1 at the tribe level (1.44: 1.36), but concentrating on *Hedysarum* indicates a reverse ratio (1.59: 1.78).

Non-canonical G-U pairing presents certain degeneracy in base-pairing which may provide structural flexibility and can be allowed within rRNA secondary structures without resulting in significant structural changes [58]. In this regard, Helices Ⅰ and Ⅲ of ITS1 and Helix Ⅳ and Ⅰ of ITS2 displayed as the most stable ones, and Helix Ⅳ of ITS1 and Helix Ⅱ ITS2 with the highest proportion of G-U bp as the most flexible ones. Subsequently, Helix 2 of the 5.8S region is the most stable, and Helix 4 is the most flexible one. Regarding the sequence variability of helices, Helix I and Ⅲ of ITS2 and Helix 4 of ITS1 *Hedysarum* possess the highest proportion of variables (variant sites/ total sites), and Helix Ⅳ of ITS2 and Helix 3 of ITS1 the lowest proportion.

Based on Bridge et al., [88], insertions/deletions in structures that affect helix length or base changes that occur in loops or bulges do not necessarily have an impact on the formation of mature functional rRNA in the ITS1 region, and these regions may be susceptible to such changes. In terms of helices length, all helices of ITS1 in clades C and H, and Helix Ⅴ of clades M and S are rendered as varied helices. In the 5.8S region lengths are fixed except for *H*. *garinense*, *Alhagi*, and most of the CEGO clade genera. However, this parameter is not profitable in the modeled ITS2 structures, structural investigation resulting from force homology modeling of ITS2DB show that Helix Ⅰ and Ⅳ have the most variable lengths. The length is the only parameter to retrieve the Helix I and Helix IV of ITS2 as the most variable helices based on Colman [61] and Zhang et al., [100], but not the helix III as the most stable one. This case is confirmed by the proportion of 100% conserved base pairs. However, the ITS1 consensus secondary structure of *Hedysarum*, specified with the lowest 100% conserved pairs displayed in Helices Ⅲ and Ⅳ, the ITS2 region with higher number distributed in helices II, III, and Ⅳ by order. This measure reaches the highest for the 5.8S region in Helices 1, 3, and 2, respectively.

### 4.4 Structure and CBC analyses of ITS1 and ITS2

The results didn’t confirm the theory reported by Coleman & Vacquier, [53]; Müller et al., [34]; and Coleman, [33] for a positive correlation between the presence of a CBC in the ITS2 secondary structure and sexual incompatibility. However, this does not mean that these organisms are the same species. Moreover, the results didn’t meet Müller et al., [34], Torres-Suárez [55], and Ozgişi [27], determining CBCs useful for generic delimitation. However, they parallel the utility of hCBCs and Nsts to distinguish species reported by Torres-Suárez, [55] and Karpenko et al., [51]. In the ITS1 region, one informative CBC is perceived between clades M and H. Although *Onobrychis* is morphologically the closer genus to the *Hedysarum*, a few species of *Onobrychis* reconstructed CBCs with three genera in the CEGO clade and *Hedysarum*.

Mostly, CBC shows its efficacy in species resolution of the preliminary organisms e. g. Protista and fungi with the ancient divergent times, the plant studies possess low cases of successful resolution. Nevertheless, comparing the nuclear divergence time of *H*. sects. *Hedysarum*, *Multicaulia* and *Stracheya* originated at 15.88, 7.92, and 4.3 Ma ago [49], and *Strychnos* (12.72 Ma) [78], lead us to the conclusion of the probability of gene-exchanging capability among members. Instead, the observed rDNA ITS1 and ITS2 secondary structural features characterized the four clades of the genus *Hedysarum* and each partly delimited the species groups and inter-specific cases. In the ITS1 structures, differences in helices lengths, bulges, and internal loops especially in helices 4 and 5, having additional helix in clade M and also hCBCs and Nsts are characteristic of the clades and species.

## Supporting information

S1 Table Intra-sectional not aligned base changes in ITS2 secondary structure of *H*. sect. *Hedysarum*. (DOCX)

S2 Table Intra-subsectional not aligned base changes in ITS2 secondary structure of *H*. sect. *Multicaulia* subsect. *Crinifera*. (DOCX)

S3 Table Intra-subsectional not aligned base changes in ITS2 secondary structure of *H*. sect. *Multicaulia* subsect. *Multicaulia*. (DOCX)

S4 Table Intra-sectional not aligned base changes in ITS2 secondary structure of *H*. sect. *stracheya*. (DOCX)

S5 Table Inter-sectional not aligned base changes in ITS2 secondary structure of *H*. sect. *Hedysarum*- *H*. sect. *Multicaulia* subsect. *Multicaulia*. (DOCX)

S6 Table Inter-sectional not aligned base changes in ITS2 secondary structure of *H*. sect. *Hedysarum*- *H*. sect. *Multicaulia* subsect. *Crinifera*. (DOCX)

S7 Table Inter-sectional not aligned base changes in ITS2 secondary structure of *H*. sect. *Hedysarum*- *H*. sect. *stracheya*. (DOCX)

S8 Table Inter-sectional not aligned base changes in ITS2 secondary structure of *H*. sect. *Multicaulia* subsect. *Multicaulia*- *H*. sect. *stracheya*. (DOCX)

S9 Table Inter-sectional not aligned base changes in ITS2 secondary structure of *H*. sect. *stracheya*- *H*. sect. *Multicaulia* subsect. *Crinifera*. (DOCX)

S10 Table Inter-subsectional not aligned base changes in ITS2 secondary structure of *H*. sect. *Multicaulia* subsects. *Multicaulia* and *Crinifera*. (DOCX)

S11 Table Inter-sectional aligned base changes in the secondary structure of 5.8S region. (DOCX)

S12 Table Diversity indices. (DOCX)
