# Supplementary material for: Characterizing nrDNA ITS1, 5.8S and ITS2 secondary structures and their phylogenetic utility in the legume tribe Hedysareae with special reference to Hedysarum
Source: PLoS One. 2023 Apr 12;18(4):e0283847. doi: 10.1371/journal.pone.0283847 (PMC10096232; doi:10.1371/journal.pone.0283847)
Supplement: S1 Table — (DOCX) [file pone.0283847.s001.docx]

**S1 Table. Intra-sectional not aligned base changes in ITS2 secondary structure of *H*. sect. *Hedysarum*.**

| 39. U A (H. campylocarpon)  47. A G (H. longigynophorum)  48. U C (H. occidentale, H. alpinumAB854491, KP338150  50. U C (H. praticola, H. wakhanicum, H. occidentale, H. alpinumAB854491, KP338150)  53. A G (H. longigynophorum)  55. U C (H. astragaloides, H. cachemirianum, H. campylocarpon, H. flavescens, H. semenovii, H. cachemirianum, H. [cuonanum](https://www.ipni.org/n/77160511-1), H. falconeri)  63. A G (H. campylocarpon)  65. U - (H. sikkimenseKY367306)  70. A G (H. dentatoalatum, H. chinense)  74. C S (H. taipeicum); C U (H. dentatoalatum, H. chinense, armenium)  80. U C (H. occidentale)  85. U C  (H. xizangensis, H. longigynophorum, H. praticola, H. wakhanicum, H. campylocarpon, H. cachemirianum, H. [cuonanum](https://www.ipni.org/n/77160511-1), H. falconeri)  90. G U (H. boutignyanum)  96. G A (H. praticola, H. wakhanicum)**;** G U (H. longigynophorum)  100. U C (H. astragaloides)  101. U C (H. semenovii, H. flavescens)  103. U A (H. longigynophorum)  104. C G (H. longigynophorum, H. xizangense)  105. U C (H. longigynophorum)  107. A G (H. polybotrysKP338182)  108. U C (H. xizangensis, H. longigynophorum)  111. U C (H. longigynophorum)  122. C U (H. campylocarpon, H. ussuriense)  123. C U (H. xizangensis, H. longigynophorum, H. praticola, H. wakhanicum, H.  jinchuanense, H. flavescens, H. semenovii, H. nagarzense, H. cachemirianum, H. [cuonanum](https://www.ipni.org/n/77160511-1), H. falconeri, H. polybotrysKP338182)  141. U - (H. austrosibiricum)  151. U C (H. algidum)  190. G C (H. sikkimenseGQ246055)  191. U G (H. astragaloides, H. cachemirianum, H. dentatoalatum, H. chinense, H. flavescens, H. semenovii); U C (H. sikkimenseGQ246055, H. algidum)  193. U C (H. sikkimenseGQ246055)  196. A G (H. flavescens, H. semenovii)  199. A U (H. wakhanicum, H. falconeri)  203. U A (H. tanguticum, H. sikkimenseGQ246055, KP338184)  212. A G (H. taipeicum)  228. C G (H. neglectum, H. hedysaroides subsp. arcticumMT081324)  231. U C (H. semenovii)  240. A U (H. ussuriense, H. sikkimenseGQ246055, H. polybotrysKP338182) |
| --- |
