# Supplementary material for: Characterizing nrDNA ITS1, 5.8S and ITS2 secondary structures and their phylogenetic utility in the legume tribe Hedysareae with special reference to Hedysarum
Source: PLoS One. 2023 Apr 12;18(4):e0283847. doi: 10.1371/journal.pone.0283847 (PMC10096232; doi:10.1371/journal.pone.0283847)
Supplement: S2 Table — (DOCX) [file pone.0283847.s002.docx]

**S2 Table. Intra- subsectional not aligned base changes in ITS2 secondary structure of *H*. sect. *Multicaulia* subsect. *Crinifera.***

| 39. C U (H. pabulare)  40. A G (H. elborsenseLC404217); A U (H. damghanicum)  45. C U (H. hyrcanicumLC404234)  49. G K (H. marandense)  52. U G (H. brachypterum)  53. A G (H. macranthum, H. micropterumLC404248); A Y (H. wrightianumLC404281, H. kalatenseQQ198826)  54. G A (H. ferganense); G U (H. sauzkenseLC404263, H. malurense, H. fallacinum (including H. longipedonculatum), H. bucharicum); G K (H. hemithamnoides)  56. G A (H. sauzkenseLC404263, H. malurense); G C (H. fallacinum (including H. longipedonculatum), H. bucharicum)  57. A G (H. micropterumQQ198817)  59. A G (H. vanenseLC404271)  60. G A (H. micropterum LC404278)  62. C G (H. damghanicum)  63. A G (H. petroviiMN871830, MN871835)  64.C U (H. gypsophylum, H. macranthum, H. micropterumLC404248, QQ198828, QQ198817, LC404278, H. wrightianumLC404282, QQ198818, QQ198819, QQ198820, QQ198821, QQ198825, H. kalatenseQQ198826, H. callithrix QQ198816, QQ198815; C N (H. wrightianumLC404281, H. kalatenseQQ198826, H. micropterum231)  69. U C (H. wrightianumLC404282, LC404279, LC404280, LC404281, ,H. callithrixKP338157, LC404208, QQ198818, QQ198819, QQ198820, QQ198821, QQ198823, QQ198824, QQ198825, H. callithrixQQ198816, QQ198815, H. gypsophylum, H. stenophyllum, H.balchanenseQQ198827, H. kalatense, H. Joharchii, H. halophillum LC404229, LC404230, LC404231, KP338167, H. macranthum, H. micropterumLC4042, QQ198828, QQ198817, LC404278; H. sp.QQ198822, H. marandense , H. volkii, H. sauzkense, H. malurense, H. renzii, H. plumosum,, H. monophyllum, H. krylovii, H. fallacinum (including H. longipedonculatum), H. kopetdaghi, H. bojnordense, H. erythroleucum, H. paucifoliolatum, H. glabrifoliolatum, H.elborsense, H. hyrcanicum, H. melanothricum, H. damghanicum, H. brachypterum) ; U Y (H. hemithamnoides, H.vanenseLC404271); U A (H. bucharicum)  70. G A (H. papillosumQQ198813)  80. U C (H. volkii)  94. G K (H. grandiflorumMT117960)  98. A G (H. anatolicum)  100. G U (H. grandiflorum, H. biebersteinii, H. sericeumLC404265 (; G K (H. eleganse, H. sericeumLC404265  101. U C (H. anatolicum, H. vanenseLC404271)  104. U C (H. borealeLC404205, H. pabulareKP338180, LC404204); U Y (H. atropatanumLC404201, LC404199)  123. C U (H. halophilumLC404231)  143. G U (H. bojnordense, H. kopetdaghiKP338172)  151. A U (H. bucharicum)  154. U C (H. damghanicum)  162. U A (H. borealeLC404205)  170. C U (H. hyrcanicumLC404234); C Y (H. criniferumLC404214)  179. C U (H. sp.QQ198822)  193. C U (H. brachypterum)  194. U C (H. damghanicum)  196. C A (H. wrightianumLC404282, LC404279, LC404280, LC404281, QQ198818, QQ198819, QQ198820, QQ198821, 220, QQ198823, QQ198824, QQ198825, H. callithrixQQ198816, QQ198815, H. gypsophylum, H. stenophyllum, H. balchanense228, H. kalatense, H. Joharchii, H. halophillumLC404229, LC404230, LC404231, KP338167, H. callithrixKP338157, LC404208, H. macranthum, H. micropterumLC404248, H. micropterumQQ198828, H. micropterum4, H. micropterum6)  199. U C (H. wrightianumLC404282, LC404279, LC404280, LC404281, QQ198818, QQ198819, QQ198820, QQ198821, QQ198822, QQ198823, QQ198824, QQ198825, H. callithrixQQ198816, QQ198815, H. Joharchii, H. kalatense, H. balchanenese228, H. gypsophylum, H. stenophyllum, H. halophillum LC404229, LC404230, LC404231, KP338167, H. callithrixKP338157, LC404208, H. macranthum, H. micropterum LC404248, H. micropterumQQ198828, QQ198817, H. micropterum6, H. marandense, H. volkii, H.sauzkense LC404264, H. renzii, H. plumosum, H. monophyllum, H. krylovii, H. kopetdaghi, H. bojnordense, H. erythroleucum, H. paucifoliolatum, H. glabrifoliolatum, H. hyrcanicum, H. melanothricumLC639678, LC639681, LC639677, LC639679, LC639680, H.elborsense LC404217, H.vanenseLC404271, H. anatolicum, H. ferganense, H. hyrcanicum LC404234, H. brachypterum, H. damghanicum, H. fallacinum (including H. longipedonculatum), H. bucharicum, H.petroviiMN871830, MN871835); U Y (H. hemithamnoides)  202. A G (H. pabulareKP338180, LC404204)  203. U W (H. persicum)  204. U G (H. petroviiMN871830, MN871835)  212. U A (H. bucharicum)  213. U G (H. boveanum subsp. europaeum)  219. C A (H. grandiflorum)  222. G A (H. bucharicum)  223. U C (H. wrightianumLC404282, LC404281, LC404280, LC404279, QQ198818, QQ198819, QQ198820, QQ198821, QQ198822, QQ198823, QQ198824, QQ198825, H. callithrixQQ198816, QQ198815, H. kalatense, H. Joharchii, H. balchanense228, H. gypsophylum, H. stenophyllum, H. halophillumLC404229, LC404230, LC404231, KP338167, H. callithrixKP338157, LC404208, H. macranthum, H. micropterumLC404248, QQ198828, QQ198817, H. micropterum LC404278)  225. C Y (H. borealeLC404205)  228. C U (H. borealeLC404205, H. pabulareKP338180, LC404204); C G (H. boveanum subsp. europaeum, H. humile)  231. U - (H. ferganense); U Y (H. garinense) |
| --- |
