# Supplementary material for: Characterizing nrDNA ITS1, 5.8S and ITS2 secondary structures and their phylogenetic utility in the legume tribe Hedysareae with special reference to Hedysarum
Source: PLoS One. 2023 Apr 12;18(4):e0283847. doi: 10.1371/journal.pone.0283847 (PMC10096232; doi:10.1371/journal.pone.0283847)
Supplement: S3 Table — (DOCX) [file pone.0283847.s003.docx]

**S3 Table. Intra- subsectional not aligned base changes in ITS2 secondary structure of *H*. sect. *Multicaulia* subsect. *Multicaulia*.**

| 46. G - (H. formosum, H. alamutense)  113. G A (H. elymaiticum)  147. A G (H. pycnostachyum, H. kotschyi)  154. C A (H. pestalozzae, H. varium, H. huetii, H. candidissimum, H. ibericum, H. pogonocarpum, H. singarense, H. syriacum)  187. U Y (H. pycnostachyum)  198. U C (H. elymaiticum, H. pestalozzae, H. varium, H. pycnostachyum, H. kotschyi, H. huetii, H. candidissimum, H. ibericum, H. pogonocarpum, H. singarense, H. syriacum)  223. A - (H. formosum, H. alamutense, H. elymaiticum, H. syriacum, H. pycnostachyum, H. kotschyi, H. huetii, H. candidissimum, H. ibericum, H. pogonocarpum, H. singarense, H. variumLC404274  225. U C (H. pestalozzaeLC404255, H. variumLC404273, LC404273)  231. U - (H. formosum, H. alamutense, H. pestalozzae, H. huetii, H. candidissimum, H. elymaiticum) |
| --- |
