# Supplementary material for: Characterizing nrDNA ITS1, 5.8S and ITS2 secondary structures and their phylogenetic utility in the legume tribe Hedysareae with special reference to Hedysarum
Source: PLoS One. 2023 Apr 12;18(4):e0283847. doi: 10.1371/journal.pone.0283847 (PMC10096232; doi:10.1371/journal.pone.0283847)
Supplement: S4 Table — (DOCX) [file pone.0283847.s004.docx]

**S4 Table. Intra-sectional not aligned base changes in ITS2 secondary structure of *H*. sect. *stracheya.***

| 47. C A (H. kumaonense)  68. G C (H. tibeticum)  143. U A (H. kumaonense)  148. C U (H. kumaonense, H. minjanense, H. wangii, H. dentatoalatum, H. cisdarvasicum) |
| --- |
