# Supplementary material for: Characterizing nrDNA ITS1, 5.8S and ITS2 secondary structures and their phylogenetic utility in the legume tribe Hedysareae with special reference to Hedysarum
Source: PLoS One. 2023 Apr 12;18(4):e0283847. doi: 10.1371/journal.pone.0283847 (PMC10096232; doi:10.1371/journal.pone.0283847)
Supplement: S5 Table — (DOCX) [file pone.0283847.s005.docx]

**S5 Table. Inter-sectional not aligned base changes in ITS2 secondary structure of *H*. sect. *Hedysarum*- *H*. sect. *Multicaulia* subsect. *Multicaulia.***

| 39. U or A C  49. G U  70. A or G G  98. U G  135. A G  137. G U  142. U G  153. G U  176. G A  195. U C  197. C A  198. C U  212. G U  228. C U  234. G A |
| --- |
