# Supplementary material for: Characterizing nrDNA ITS1, 5.8S and ITS2 secondary structures and their phylogenetic utility in the legume tribe Hedysareae with special reference to Hedysarum
Source: PLoS One. 2023 Apr 12;18(4):e0283847. doi: 10.1371/journal.pone.0283847 (PMC10096232; doi:10.1371/journal.pone.0283847)
Supplement: S6 Table — (DOCX) [file pone.0283847.s006.docx]

**S6 Table. Inter-sectional not aligned base changes in ITS2 secondary structure of *H*. sect. *Hedysarum*- *H*. sect. *Multicaulia* subsect. *Crinifera*.**

| 39. A or U C  47. A or G A  64. C C or U  70. G or A G  96. A or G or U A  97. U A  100. U or C C or U  142. U U or G  153. A U  154. G U  190. G or C A  191. (G or U or C) G  195. U A or C  199. C C or U or Y  212. A U  228. C U  234. G A |
| --- |
