# Supplementary material for: Characterizing nrDNA ITS1, 5.8S and ITS2 secondary structures and their phylogenetic utility in the legume tribe Hedysareae with special reference to Hedysarum
Source: PLoS One. 2023 Apr 12;18(4):e0283847. doi: 10.1371/journal.pone.0283847 (PMC10096232; doi:10.1371/journal.pone.0283847)
Supplement: S7 Table — (DOCX) [file pone.0283847.s007.docx]

**S7 Table. Inter-sectional not aligned base changes in ITS2 secondary structure of *H*. sect. *Hedysarum*- *H*. sect. *stracheya.***

| 64. C A  96. G A  184. A G  211. C U  212. A U  234. G A |
| --- |
