# Supplementary material for: Characterizing nrDNA ITS1, 5.8S and ITS2 secondary structures and their phylogenetic utility in the legume tribe Hedysareae with special reference to Hedysarum
Source: PLoS One. 2023 Apr 12;18(4):e0283847. doi: 10.1371/journal.pone.0283847 (PMC10096232; doi:10.1371/journal.pone.0283847)
Supplement: S8 Table — (DOCX) [file pone.0283847.s008.docx]

**S8 Table. Inter-sectional not aligned base changes in ITS2 secondary structure of *H*. sect. *Multicaulia* subsect. *Multicaulia*- *H*. sect. *stracheya*.**

| 39. C U  47. G C  50. U G  65. C A  71. G A  99. G U  104. U C  108. G A  109. C U  136. G A  137. U G  143. G U  152. C U  154. U G  155. C A  177. A G  185. A G  191. A G  196. A C  197. C A  198. C U  199. U C  212. C U  228. U C |
| --- |
