# Supplementary material for: Characterizing nrDNA ITS1, 5.8S and ITS2 secondary structures and their phylogenetic utility in the legume tribe Hedysareae with special reference to Hedysarum
Source: PLoS One. 2023 Apr 12;18(4):e0283847. doi: 10.1371/journal.pone.0283847 (PMC10096232; doi:10.1371/journal.pone.0283847)
Supplement: S9 Table — (DOCX) [file pone.0283847.s009.docx]

**S9 Table. Inter-sectional not aligned base changes in ITS2 secondary structure of *H*. sect. *stracheya* - *H*. sect. *Multicaulia* subsect. *Crinifera*.**

| 39. U C  64. A U or C or Y  70. A G  97. U A  107. A G  152. A U or C  153. G U  185. G A  191. G A  196. U C  228. U C |
| --- |
