# Supplementary material for: Characterizing nrDNA ITS1, 5.8S and ITS2 secondary structures and their phylogenetic utility in the legume tribe Hedysareae with special reference to Hedysarum
Source: PLoS One. 2023 Apr 12;18(4):e0283847. doi: 10.1371/journal.pone.0283847 (PMC10096232; doi:10.1371/journal.pone.0283847)
Supplement: S10 Table — (DOCX) [file pone.0283847.s010.docx]

**S10 Table. Inter-subsectional not aligned base changes in ITS2 secondary structure of *H*. sect. Multicaulia subsects. Multicaulia and *Crinifera*.**

| 49. G U  64. C or U or Y C  68. G or U G  69. U or C or A or Y C  98. A or G U  99. U G  101. U or C U  104. U or C or Y U  109. U C  136. A G  138. G U  143. G or U G  148. A A or G  152. U C  153. U A  162. U or A U  177. G A  197. A C  198. C A  212. U or A C |
| --- |
