# Supplementary material for: Characterizing nrDNA ITS1, 5.8S and ITS2 secondary structures and their phylogenetic utility in the legume tribe Hedysareae with special reference to Hedysarum
Source: PLoS One. 2023 Apr 12;18(4):e0283847. doi: 10.1371/journal.pone.0283847 (PMC10096232; doi:10.1371/journal.pone.0283847)
Supplement: S11 Table — (DOCX) [file pone.0283847.s011.docx]

**S11 Table. Inter-sectional aligned base changes in the secondary structure of 5.8S region.**

| 1. A G (H. renzii, H.tanguicum, H. sikkimense); A R (H.criniferumLC404214)  2. A U, W (H. hyrcanum)  5. C U (clade M, and clade C except H. damghanicum); C Y (H. marandense45)  14. G R (H. garinense, H. sauzakenseLC404264)  130. A U (H. xizangensis, H. cuonanum, H.boutignianum, H. campylocarpon, H. falconeri, H.praticolum, H. wakhanicum, H. astagaloides, H. longigynophorum, H, cachemirianum)  133. A U (H. xizangensis) |
| --- |
