# Supplementary material for: Characterizing nrDNA ITS1, 5.8S and ITS2 secondary structures and their phylogenetic utility in the legume tribe Hedysareae with special reference to Hedysarum
Source: PLoS One. 2023 Apr 12;18(4):e0283847. doi: 10.1371/journal.pone.0283847 (PMC10096232; doi:10.1371/journal.pone.0283847)
Supplement: S12 Table — (DOCX) [file pone.0283847.s012.docx]

| **S12 Table. Diversity indices.** | | | | | | | | | | | | |
| --- | --- | --- | --- | --- | --- | --- | --- | --- | --- | --- | --- | --- |
| **Tribe Data set** | **Polymorphic sites** | **Par. inf. sites** | **Singletone Sites** | **Num. of haplo.** | **Indel Sites analysed** | **Indel Haplo.** | **indel haplo. Div.** | **Eta** | **Theta**  **from Eta** | **Nuc. diversity )Pi(** | **Haplotype diversity** | Tajima's D |
| **ITS1** | 102 | 72 | 30 | 68 | 32 | 27 | 0.604 | 152 | 0.15498 | 0.06598 ± 0.00385 | 0.918 ± 0.00015 | -1.79924 |
| **5.8S** | 8 | 3 | 5 | 8 | 0 | 0 | 0 | 8 | 0.00879 | 0.00186 ± 0.00039 | 0.230 ± 0.039 |  |
| **ITS2** | 93 | 58 | 35 | 70 | 42 | 21 | 0.811 | 134 | 0.12297 | 0.05620± 0.00287 | 0.910 ± 0.014 | -1.90783 |
| **ITS** | 212 | 142 | 70 | 93 | 72 | 34 | 0.851 | 305 | 0.10134 | 0.04637 ± 0.00235 | 0.947 ± 0.010 |  |
